# The experience of cognitive behavioural therapy in depressed adolescents who are fatigued

**DOI:** 10.1111/papt.12365

**Published:** 2021-09-21

**Authors:** Georgia Tanith Herring, Maria Elizabeth Loades, Nina Higson‐Sweeney, Emily Hards, Shirley Reynolds, Nick Midgley

**Affiliations:** ^1^ Department of Psychology University of Bath UK; ^2^ Bristol Medical School University of Bristol UK; ^3^ School of Psychology and Clinical Language Studies University of Reading UK; ^4^ Research Department of Clinical, Educational and Health Psychology University College London UK; ^5^ Child Attachment and Psychological Therapies Research Unit (ChAPTRe) Anna Freud National Centre for Children and Families London UK

**Keywords:** fatigue, depression, major depressive disorder, cognitive behavioural therapy, adolescents

## Abstract

**Objective:**

Fatigue is a common and debilitating symptom of major depressive disorder (MDD). Cognitive behavioural therapy (CBT) is a recommended psychological treatment for adolescents with moderate to severe depression. This study explored the experience of CBT in fatigued adolescents with MDD.

**Design:**

A qualitative study was conducted using existing data from the qualitative arm of a large randomized control trial, the IMPACT study.

**Methods:**

Data were obtained from semi‐structured interviews conducted after therapy. Participants were 18 adolescents (aged 13–18 years) who reached the clinical threshold for fatigue on diagnostic assessment before starting treatment. The data were analysed using thematic framework analysis.

**Results:**

Three themes and seven sub‐themes were developed. Adolescents appeared to find taking part in initial sessions, engaging in ongoing sessions and completing homework challenging. Perceiving the therapist as genuine seemed to provide a sense of safety which enabled adolescents to open up in sessions. When the therapist was not perceived as genuine, adolescents appeared to find CBT less helpful. The structure of CBT appeared to enable treatment goals to be set, and facilitated an increase in meaningful activity. Ensuring that tasks were perceived as manageable and goals as achievable seemed important for participation. Cognitive restructuring appeared useful, although some adolescents tended to engage in distraction from thoughts as an alternative strategy.

**Conclusions:**

This study provides an initial insight into how fatigued adolescents with MDD experience CBT. Further research is required to establish whether the themes are pervasive and relatedly, how best to treat depression in fatigued adolescents receiving CBT.

**Practitioner points:**

- Fatigued adolescents with depression found engaging in CBT sessions and therapeutic homework demanding.
- Establishing a collaborative therapeutic relationship, where the therapist was perceived as genuine, appeared helpful for participation.
- The structured approach to therapy, combined with flexibility, was experienced as helpful. Adolescents who perceived the pace of sessions to be manageable and therapeutic goals as achievable seemed to find CBT helpful overall.
- These findings provide insight into how fatigued adolescents with depression experience CBT and highlight the importance of being aware of fatigue and adapting therapy accordingly.

## Background

Depression is a leading cause of illness and disability among adolescents worldwide (Word Health Organization, 2021). A diagnosis of Major Depressive Disorder (MDD) places adolescents at an increased risk of suicide (Mullen, 2018), reduced social functioning (Verboom, Sijtsema, Verhulst, Penninx, & Ormel, 2014) and recurrent MDD in adulthood (Rohde, Lewinsohn, Klein, Seeley, & Gau, 2013). A randomized controlled trial (RCT) comparing psychological treatments for adolescents with MDD, the IMPACT study (Goodyer et al., 2017), conducted nested qualitative research (Midgley, Ansaldo, & Target, 2014) to explore adolescents’ experiences of depression (Midgley et al., 2015) and their expectations and experiences of therapy (Midgley et al., 2016). This is important because adolescent presentations of MDD can differ from adults (Roberts, 2013). For example, adolescents with MDD are more likely to present with irritable mood (Powell, Ocean, & Stanick, 2017) and communicate somatic complaints (Bohman, 2012). These differences in presentation and phenomenology may have implications for how depression and treatments for depression are experienced by adolescents.

Fatigue is a somatic symptom of depression which is commonly reported by adolescents with MDD (Nardi, Francesconi, Catena‐Dell'osso, & Bellantuono, 2013; Orchard, Pass, Marshall, & Reynolds, 2017). In the IMPACT study cohort of 465 participants, 73.3% of adolescents with a primary diagnosis of MDD experienced significant fatigue (Goodyer et al., 2017). The Diagnostic and Statistical Manual of Mental Disorders, Fifth Edition presents the diagnostic features of fatigue in MDD as a decrease in energy, tiredness, a reduced efficacy in tasks which are accomplished and complaints of fatigue without physical exertion (American Psychological Association [APA], 2013). Similarly, the International Statistical Classification of Diseases and Related Health Problem, tenth revision (ICD‐10) includes ‘decreased energy or an increased fatiguability’ as one of the core criteria for mild to severe depression (World Health Organization, 1993, pp. 82–83).

Psychological research has conceptualized fatigue as a subjective and debilitating symptom (Fava et al., 2014; Jason, Evans, Brown, & Porter, 2010). Fatigue severity has been associated with increased cognitive and functional impairment, elevated risk of suicide, and increased anxiety in university students with significant depressive symptoms (Nyer et al., 2015). Additionally, ter Wolbeek, van Doornen, Kavelaars, and Heijnen (2008) reported adolescents who were persistently fatigued had more symptoms of depression and anxiety, were less physically active and slept fewer hours than non‐fatigued adolescents. Furthermore, fatigue is one of the most common residual symptoms in adults with remitted depression post‐treatment (Baldwin & Papakostas, 2006; Conradi, Ormel, & De Jonge, 2011), is a risk factor for depressive chronicity (Moos & Cronkite, 1999) and predicts an inabiliy to achieve remission with treatment (Fava et al., 2014). Thus, fatigue appears to be a prevalent and disabling symptom of MDD, which may place adolescents under an increased risk of adverse outcomes if experienced persistently, or at a significant level.

Both fatigue and depression are complex, heterogeneous constructs. Arnold (2008) highlighted the conceptual similarities between fatigue and depression, noting that physical, affective and cognitive dimensions of fatigue, including decreased activity, low motivation, and reduced concentration and mental endurance are present in other criteria of MDD. These similarities in symptomology can make it difficult to distinguish between fatigue and depressive syndromes (Arnold, 2008). Derived from the medical model, depression is viewed as a distinct illness with defined symptomology (Clark, Cuthbert, Lewis‐Fernández, Narrow, & Reed, 2017). Although individuals who are diagnosed with depression may present with shared characteristics, there are many different symptom combinations that meet the diagnostic criteria (Fried & Nesse, 2015). Additionally, each symptom may be experienced in a different way and to a different degree. For example, fatigue as a depressive symptom can be viewed to exist on a continuum, from its absence to a severely impactful presence. This multi‐dimensionality of depression means that the structure of classification systems can present challenges in the way we conceptualize MDD.

Even though it is a common symptom in adolescents who are depressed, fatigue may not be thoroughly assessed in Child and Adolescent Mental Health Services (CAMHS) and does not tend to be prioritized, with clinicians often focussing on sleep problems rather than fatigue (Higson‐Sweeney, Loades, Hiller, & Read, 2020). This means fatigue may be overlooked in clinical settings, despite how common it is and how debilitating it can be.

Cognitive behavioural therapy (CBT) is a recommended treatment for moderate to severe depression in young people aged 12–18 years (National Institute for Health & Care Excellence [NICE], 2019). CBT is effective in reducing depressive symptoms in adolescents (Keles & Idsoe, 2018; Weersing, Jeffreys, Do, Schwartz, & Bolano, 2017) and in reducing the risk of depression persisting at 17–19 weeks follow up by 63% in adolescents with sub‐clinical depressive symptoms (Oud et al., 2019). Furthermore, the IMPACT study provided evidence of CBT’s long‐term effectiveness, as reductions in depressive symptoms were maintained at 86 weeks (Goodyer et al., 2017).

CBT is a structured and goal‐orientated therapy which focuses on the client’s presenting problem and the processes which are maintaining their difficulties (Kennerley, Kirk, & Westbrook, 2017). In CBT, clients work together with the therapist and are active participants, both within therapy sessions and by applying skills learned in therapy between sessions (Fenn & Byrne, 2013; Tang & Kreindler, 2017). Establishing a collaborative therapeutic relationship and taking an active role in their treatment is an important part of adolescent engagement in CBT (Donnellan, Murray, & Harrison, 2013). The cognitive component of CBT encourages adolescents to identify and challenge their dysfunctional beliefs and negative automatic thoughts, and to consider how to replace negatively biased cognitions with neutral or positive interpretations (Brewin, 2006). The behavioural component of CBT typically focuses on increasing activities that lead to experiences of mastery and pleasure and managing inter‐personal relationships through social problem‐solving (Kazdin & Weisz, 1998). Adolescents receiving CBT for depression have reported that it helped them by increasing their awareness of how they interpreted situations and by encouraging them to do more pleasurable activities (Bru, Solholm, & Idsoe, 2013).

It is possible that adolescents with MDD who are fatigued could experience the therapeutic process and requirements of CBT as particularly demanding. CBT requires adolescents to attend sessions and actively engage in their treatment by working collaboratively with the therapist and implementing therapeutic techniques (Cully & Teten, 2008). However, the effects of fatigue (e.g., low energy) could negatively impact the experience of CBT. Depressed adolescents have reported fatigue as interfering with engagement in brief behavioural activation (Watson, Harvey, Pass, McCabe, & Reynolds, 2021), so it is possible this could extend to CBT. Exploring how fatigue may affect adolescents’ experience of psychological treatments could highlight ways in which components of therapy could be adapted to meet the needs of these adolescents.

This qualitative study aimed to understand how CBT is experienced by depressed adolescents with clinically significant fatigue. Qualitative research seeks to explore and understand the in‐depth meaning and complexities of social contexts and phenomenon (Guest, Namey, & Mitchell, 2013). Thus, it was deemed an appropriate method by which to explore adolescents’ therapeutic experiences.

## Methods

### Setting for the study

A secondary data analysis was conducted using semi‐structured interview data from the Improving Mood with Psychoanalytic and Cognitive Therapies: My Experience (IMPACT‐ME) study (Midgley et al., 2014). The IMPACT‐ME study was a longitudinal qualitative study nested within the IMPACT RCT (Goodyer et al., 2011, 2017). The IMPACT RCT compared the effectiveness and cost‐effectiveness of CBT, short‐term psychoanalytic psychotherapy and a brief psychosocial intervention in treating MDD in clinically referred adolescents (*N* = 465; Goodyer et al., 2017). IMPACT‐ME conducted interviews with a sub‐group of participants (*n* = 77), who were receiving their assigned treatment from a CAMHS in North London (Midgley et al., 2014). The interviews were conducted at three time points: pre‐treatment (baseline), post‐treatment (36 weeks) and follow up (86 weeks; Midgley et al., 2014). MDD diagnosis was confirmed at baseline using the Kiddie Schedule for Affective Disorders and Schizophrenia (K‐SADS; Kaufman et al., 1997). This study will analyse the post‐treatment interview data from the adolescents who took part in the IMPACT‐ME sub‐study, were allocated to the CBT treatment arm and obtained clinical threshold fatigue on the K‐SADS at baseline.

### Sample and recruitment

Clinical staff at CAMHS screened patients’ eligibility for the IMPACT study. The inclusion criteria consisted of being aged 11–17 and having a current DSM‐IV diagnosis of MDD, with moderate to severe impairment. The exclusion criteria consisted of a primary diagnosis of Schizophrenia, Bipolar I or an eating disorder; having diagnosed learning difficulties or a pervasive developmental disorder; the taking of medication which may disrupt selective serotonin reuptake inhibitors; and an inability to cease taking the medication, substance abuse and pregnancy (Goodyer et al., 2011). On agreeing to participate in the IMPACT study, informed consent was obtained from the adolescent and their primary caregiver (Goodyer et al., 2011).

The transcripts that were analysed in this secondary data analysis were selected from a sample of 41 adolescents, who were randomized to the CBT arm of the IMPACT trial and were interviewed as part of the IMPACT‐ME sub‐study. The post‐treatment interview transcripts of those adolescents who obtained a clinical threshold score of fatigue on the K‐SADS at baseline (*n* = 18) were provided by the Anna Freud Centre for analysis. All identifiable material was removed, and names were replaced with pseudonyms. The sample consisted of female (*n* = 12) and male (*n* = 6) adolescents aged 13–18 years (mean = 15.83; Table 1). The adolescents attended a mean number of 10 sessions, with 11 having come to an agreed ending with their therapist. Seven adolescents ended therapy without their therapist's agreement and were considered non‐completers. The mean number of sessions attended is comparable to the figures for the overall IMPACT study, as is the proportion who can be considered non‐completers (Goodyer et al., 2017). However, previous analysis of data from the IMPACT study has shown that non‐completers were themselves a heterogenous group. Although in some cases non‐completion was associated with poorer outcomes, that was not always the case (O’Keeffe et al., 2019; O’Keeffe, Martin, Target, & Midgley, 2019). Participants in this sample were recruited from North London, a densely populated area which inhabits approximately four million people. These participants were more ethnically diverse than participants recruited to the trial in other parts of England, which included East Anglia and the North West of England. East Anglia inhabits approximately three million people in its largely rural areas and 100,000 people in four urban areas. North West England is inhabited by approximately four million people, one million of these inhabit rural areas and three million reside in the city of Manchester (Goodyer et al., 2017).

**Table 1:** Participant characteristics of adolescents assigned to the CBT treatment arm and who scored clinical threshold fatigue on the KSADS at baseline

Pseudonym	Gender	Age	Ethnicity	Therapy attendance
Tia	Female	13	White British	Non‐completer
Ella	Female	16	White and Black African	Non‐completer
Amira	Female	17	Bangladeshi	Completer
Rachel	Female	15	Other Black background	Completer
Mia	Female	16	White British	Completer
Peyton	Female	16	White and Black Caribbean	Non‐completer
Jess	Female	17	White British	Non‐completer
Olivia	Female	17	Bangladeshi	Completer
Phoebe	Female	14	White British	Completer
Katie	Female	17	Other mixed background	Completer
Milly	Female	15	White British	Completer
Anya	Female	17	Other White background	Non‐completer
Mike	Male	18	White British	Non‐completer
Ben	Male	14	White British	Completer
Harry	Male	17	Other White background	Completer
Finn	Male	14	White and Asian	Non‐completer
Rob	Male	16	White British	Completer
Taylor	Male	16	Other Asian background	Completer

### Data collection

Interviews were conducted by one of a team of trained research assistants and took place at the participant’s home or at the CAMHS clinics, depending on the adolescent’s preference. The post‐treatment interview was the ‘Experience of therapy’ interview (Midgley et al., 2011), which explored the adolescents’ experience of therapy, perceived changes and how they understood what had contributed to any of the changes that they described. The participants were encouraged to share their experience in their own words and were reminded that there were no right or wrong answers (Midgley et al., 2016). For further information regarding the interview topic guides see Midgley et al. (2014).

### Ethical considerations

This study used secondary data from pre‐approved research: The IMPACT study was granted approval from Cambridgeshire Research Ethics Committee (REC Reference: 09/H0308/137; Goodyer et al., 2011). Additionally, the Psychology Research Ethics Committee from the psychology department at the University of Bath approved the proposed secondary data analysis (PREC reference: 20‐093).

### Data analysis

Framework analysis is a qualitative method which was initially developed for social policy research (Ritchie & Spencer, 1994) and has been used in a variety of research disciplines (Parkinson, Eatough, Holmes, Stapley, & Midgley, 2016; Ward, Furber, Tierney, & Swallow, 2013). Framework analysis sits within the thematic methodology (Gale, Heath, Cameron, Rashid, & Redwood, 2013) and was deemed an appropriate method as it enabled deductive and inductive approaches to analysis. A deductive approach was required when selecting data from the transcripts which related to the adolescents’ experience of therapy. An inductive approach was taken when coding the data, enabling data‐driven themes to be developed. Codes were predominantly semantic to capture the participants’ explicit meaning. Latent codes were assigned to data when researcher GH interpreted a deeper underlying meaning.

Data interpretation was underpinned by a constructivist position: adolescents constructed their realities through their subjective therapeutic experiences and GH constructed her knowledge of the adolescents’ experiences through her interpretation of the data (Lincoln & Guba, 2013).

To ensure a reflexive approach to analysis, GH sought feedback from post‐graduate student colleagues NH‐S, JM and KS and sought input from academic supervisors to refine the themes (ML and EH). GH met with co‐authors ML and EH regularly, which promoted reflection. GH, JM and KS were naïve to qualitative research, with experience from academic study. ML, EH and NH‐S have qualitative research experience. ML is a clinical psychologist with experience in CAMHS settings and was a CBT therapist in the IMPACT study. GH sought to be cognisant of prior assumptions brought to data analysis by herself and student colleagues by discussing interpretations of the data with colleagues at meetings. The following stages of analysis were conducted as outlined by Gale et al. (2013; Table 2).

**Table 2:** Stages of framework analysis which were conducted in this secondary data analysis

Stage of analysis	How the stage was implemented	Persons involved in stage of analysis
Data Familiarization	Transcripts read and re‐read Fatigue terms ‘tired, energy, worn out, bothered and fatigue(ed)’, were searched, ensuring relevant data were not missed Relevant sections of transcripts were exported into Microsoft Excel	GH conducted this for all transcripts KS and JM repeated this separately on two transcripts and GH compared this
Coding	Codes were developed and refined After receiving feedback, codes that did not accurately reflect the data were amended	GH developed initial codes KS, JM and GH discussed initial codes via a video meeting The codes were refined by GH NH‐S reviewed 50% of the coded data
Developing an analytical framework	Refined codes were grouped into categories which represented the coded data Saturation was deemed to be achieved: no new concepts emerged from the data that were not represented by an existing category (Suter, 2012)	GH developed the analytical framework
Applying the analytical framework	Data were indexed by category using the sort and filter tool in Excel Each category yielded a column of related data and cases related to the category could be identified by row Feedback ensured the categories encompassed the coded data	GH applied the analytical framework NH‐S reviewed the first five rows of coded data assigned to each category
Charting the data	Data from each category and each participant were summarized Charting refined data into sizeable chunks and enabled connections and patterns to be identified	GH charted the data
Data Interpretation	Connections between categories were identified Priori concepts or concepts that emerged from the data were explored Themes were developed using a spreadsheet where illustrative quotes from related categories were allocated by column	GH developed initial themes GH discussed initial themes with NH‐S, JM and KS Themes were shared with ML and EH for their input

## Results

**Figure 1:**
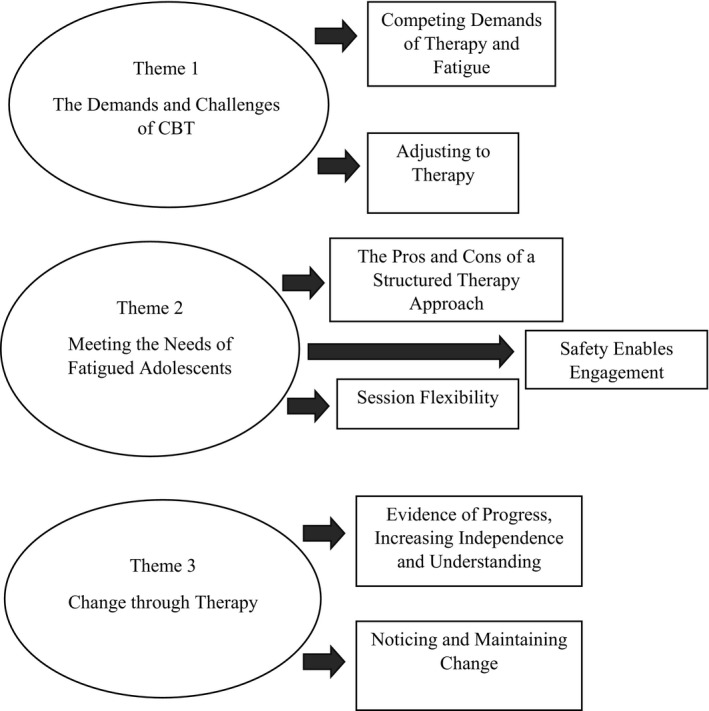
Thematic diagram illustrating themes and sub‐themes.

Three themes and seven sub‐themes were developed using framework analysis (Figure 1). The themes were created to encompass salient narratives that represented the adolescents’ experience of CBT. Each theme has been described below with illustrative quotes.

### Theme 1: The demands and challenges of CBT

This theme encapsulates the difficulties adolescents experienced during therapy. The first sub‐theme ‘Competing demands of therapy and fatigue’ explores how the difficulties adolescents experienced appeared to stem from the demands of therapy and fatigue. These difficulties included the ways adolescents struggled to engage in sessions and complete therapeutic homework. The second sub‐theme ‘Adjusting to therapy’ encapsulates adolescents’ initial uncertainty and difficulty coping with the emotions evoked during early therapy sessions.

#### Competing demands of therapy and fatigue

Adolescents described various difficulties taking part in their treatment. For some, attending therapy presented challenges which they strived to overcome. Amira (17) described struggling to get out of bed to attend her sessions:

…it was more forcing myself to get out of bed not the actually going part…if it was cold and raining and just you know I felt weak anyway physically…but I still battled through that and went…

Engaging in sessions appeared particularly challenging for adolescents. Anya (17) described therapy as ‘emotionally draining’ and spoke about her difficulty engaging in sessions as she struggled to concentrate:

I couldn’t concentrate…she could tell when my eyes were drifting off… I could listen for literally like 20 minutes at most

For some participants, engaging in sessions appeared stressful ‘I had to like repeat what I was like goin through again and again and again…’ (Tia, 13). ‘I kind of just wanted to be over and done with it so I could relax’ (Ella, 16). At times, adolescents lacked motivation to engage in therapy ‘I didn’t wanna work’ stated Milly (15) ‘if you’re feelin low and you don’t particularly want to talk’.

Difficulties taking part in treatment appeared to extend outside of sessions. Phoebe (14) described the demands of documenting her activity log which appeared to require contemplation and commitment ‘after you did something you kind of have to remember to write it down and then think‐thinking over it and then how you felt and then say putting it into a number’.

For others, engaging in therapeutic homework appeared overwhelming. When asked to keep a mood diary in between sessions, Tia (13) described thinking ‘I’m never gonna do this, I just, I’m just wasting this lady’s time and my own time’. Milly (15) described how she struggled to complete therapeutic homework alongside her schoolwork, which appeared to impact subsequent sessions:

It was making me feel worse because he was giving me homework that I didn’t do. And then like when it comes to the homework part of the next session… have you done it? I’ll be like no. Have you got it with you? No…I don’t feel like it helps me in any way.

#### Adjusting to therapy

Adolescents appeared to find their initial sessions particularly challenging, often communicating uncertainty and apprehension. ‘I just started crying’ explained Ben (14) as he described the worries he experienced. ‘I was sort of worried about what was gunna happen, stressed about what was gunna happen… just wondering what was gunna happen really’.

Adolescents appeared to adjust to the therapeutic dynamics which may have enabled them to emotionally open up. Katie (17) described how initially she was ‘quite closed’ but once she began to confide in her therapist she felt ‘relief was coming off my shoulders’.

However, for some adolescents, describing their difficulties in sessions brought further difficulty. ‘It just kind of felt like it was kind of bringing it all…say to the surface again…’ (Phoebe, 14).

Anya (17) metaphorically described opening up in the initial sessions to creating an ‘open wound’, which she did not have the self‐management skills to deal with and was not yet the focus of the sessions:

they're like leaving you… without like help like you know it’s kind of like they're not like helping like stitch the wound up they're just kind of like leaving it open…

However, once the focus of the sessions appeared more proactive, adolescents may have perceived therapy as more helpful.

about… a quarter of the way through…when we kind of stopped talking about everything…and instead thinking of ways to deal with it and cope with it I think that's kind of when I realised that… it was actually… probably gonna help (Phoebe, 14).

### Theme 2: Meeting the needs of fatigued adolescents

Fatigued adolescents’ experiences of CBT seemed to largely depend on how well CBT met their specific needs and symptoms. The sub‐themes generated reflect three core components of CBT which appeared most impactful. The first sub‐theme, ‘The pros and cons of a structured therapy approach’ highlights how in the context of fatigue, the structure of CBT, including how therapeutic goals and activities were experienced could be helpful or a hindrance for adolescents. The second sub‐theme, ‘Safety enables engagement’, presents the importance of the therapeutic alliance for engagement, and the consequences for adolescents if there is a lack of rapport. The third sub‐theme, ‘Session flexibility’, considers how session frequency and length contributed to difficulties in engagement, and the need for increased flexibility to meet fatigued adolescents’ needs.

#### The pros and cons of a structured therapy approach

For some, a structured approach to treatment appeared to enable the therapist and adolescent to identify and tackle key issues. Mike (18) expressed having a ‘clear goal’ gave him ‘a sense of ya’know, what I’m doing wiv this, wiv this time’. Olivia (17) also appeared to find the structured session content helpful, which may have made her feel supported in her treatment ‘I like the fact that it had more structure to it…I guess it’s like they were aware of the changes that should happen’.

A structured approach to activity may have helped adolescents combat fatigue symptoms. Amira (17) highlighted the importance of her therapist structuring her activity log at her pace ‘building up the ability and the strength to physically do them and then also the sort of mental stamina to think yeah I'm gonna do this’.

However, there were ways in which CBT’s structured approach did not meet adolescents’ needs ‘there’s already a system laid out for it and you’re just kind of jumping through hoops…like it’s not individual to your situation’ (Mike, 18). Anya (17) felt the structure of therapy was prioritized and lacked collaboration, perhaps to the extent that it hindered her treatment:

‘I don't think she ever cared about me…I think she cared more about the CBT and like keeping to the structure’ as she explained how she felt unable to complete therapeutic tasks ‘I just wasn’t ready to do any of the steps’.

#### Safety enables engagement

Another way CBT appeared to meet the adolescents’ needs was by providing an environment where they felt listened to and valued ‘it was important that I went somewhere where someone would listen to me and think that I was significant…’ (Olivia, 17).

The relationship with the therapist seemed important for adolescents to engage in therapy. Katie (17) described how her therapist’s non‐judgmental stance was particularly helpful:

I started feeling more comfortable…the way that she was talking to me I didn’t feel like she was judging me…

For some, therapy seemed to provide a sense of reassurance regarding their diagnosis. In Amira’s (17) case, the reassurance she received may have helped tackle self‐blame:

They sort of reassured me too… this is a REAL thing, that is an illness and some people have it and it’s awful and it isn’t your fault… to hear that was important…

However, others did not feel that therapy provided them with a congenial environment. Ella (16) described the therapeutic environment as ‘too professional’ which may have hindered her engagement, ‘if she made it a bit more comfortable… and a bit more relaxed then maybe I would have felt more comfortable in talking to her’.

Milly (15) described how she felt her therapist was ‘listening, but not listening’. These adolescents appeared to perceive a lack of genuineness in their therapist or the therapeutic environment which did not provide them with a sense of safety and therefore may not have met their needs as clients.

#### Session flexibility

The ability for sessions to be flexible appeared important to adolescents. ‘I have to go obviously for my well‐being but sometimes I wish that the schedule was a bit more flexible’ (Katie, 17). Session length was one component which appeared to impact on their therapeutic experience. Ella (16) described ‘looking at the clock to see when it was over… it was like 50 minutes, it’s quite long’. However, Anya (17), who struggled with her concentration during sessions, explained how she and her therapist shortened her sessions ‘which really helped coz… I felt like, ‘oh thank god!’, it wasn’t too much in one go’.

Session frequency also appeared to be a component which adolescents felt could have been made more flexible. Olivia (17) suggested a longer gap between sessions would have enabled her to implement therapeutic techniques and bring more to sessions ‘I would just have more time to put stuff into action…like there would be more to tell her…’.

Rob (16) explained how a flexible approach was taken to ensure his rest day was not interrupted ‘I should have had a session then but we changed it to be less frequently, so it didn’t interrupt with that [rest] day’.

### Theme 3: Change through therapy

This theme consists of two sub‐themes. The first sub‐theme ‘Evidence of progress, increasing independence and understanding’ encompasses how adolescents appeared to find evidence of their progress in therapy, independence in treatment and gaining an understanding of themselves or others as key components to change. The second sub‐theme ‘Noticing and maintaining change’ describes the changes adolescents’ perceived since completing CBT, including how they utilized the skills learned in therapy and whether they experienced changes in fatigue.

#### Evidence of progress, increasing independence and understanding

Adolescents appeared to find being presented with evidence of their progress particularly helpful during therapy. Rachel (15) reflected on her therapeutic homework which provided her with evidence of the behaviours she had implemented, which she seemed to find rewarding:

It’d help to like read it, just like, if somethings bad, I can see, I can do something better, if somethings good, then yeah I could do that again…and like, be proud that I did it in a certain way…

CBT appeared to encourage adolescents to facilitate change independently. Finn (14) described how CBT provided him with ‘a lot of ideas and solutions on like how to help’ which he was able to implement himself. Olivia (17) appeared to find engaging in activities in between sessions provided her with a sense of independence in treatment.

I became sort of more active because like it made sure that there’s an actual link between being… between therapy and the rest of my life… so that's how I could actually make an impact…

Adolescents also appeared to perceive change through therapy by gaining a deeper understanding of themselves and others ‘this whole thing helped me understand other people as well… so, therapy did help in that sense’ (Taylor, 16). While Katie (17) described gaining insight into herself ‘every time I have a session, I feel like I learnt something new about myself’.

##### Noticing and maintaining change

Adolescents appeared to perceive change through therapy ‘I looked so zombie‐like but now it’s like complete transition like within like 6 months’ (Anya, 17). However, others perceived less change, particularly regarding fatigue‐related problems ‘I am still always tired’ (Taylor, 16).

Most adolescents expressed that CBT had equipped them with skills to manage their thoughts and feelings.

she’s given me skills like breathing skills, thinking skills…the type of skills to, to cope with it and to understand it so that I can get through it rather than letting it take over my life again (Phoebe, 14).

However, others communicated difficulties maintaining the changes they had made. Rachel (15) described how during therapy she was able to go to the shops again ‘I felt, basically like invincible’, but struggled to maintain her progress ‘it started going back to how it was’.

One self‐management method adolescents used was to distract themselves. Harry (17) described how when he began to feel low, he would call a friend or take a shower ‘I'd just be like in another world’. While Finn (14) would ‘listen to music’ when he felt ‘angry or upset’. Similarly, Phoebe (14) expressed a tendency to engage in distraction rather than utilize therapeutic skills:

even though I'd be able to manage them it would just kind of… I wouldn’t really wanna think about it… I just like turn the tv on or get the laptop…so I'm occupied

## Discussion

When asked about their experiences of CBT, adolescents with depression who also reported fatigue did not tend to explicitly discuss fatigue, but fatigue appeared to impact their therapeutic experience in nuanced ways. Additionally, the therapeutic process and requirements of CBT appeared challenging. Adolescents described struggling with the emotions that were provoked in early sessions, engaging in ongoing sessions and completing therapeutic homework.

Adolescents found taking part in CBT sessions demanding and some were reluctant or felt unable to discuss their difficulties. Difficulties engaging in therapy are common in adolescents who may not have sought treatment themselves and enter therapy with varying levels of cooperativeness (Bolton Oetzel & Scherer, 2003; Curry & Reinecke, 2003). Participants described struggling to engage in therapy due to their low mood or because they found sessions stressful. Although it is unclear whether engagement was impacted by fatigue, participants who explicitly talked about fatigue in their accounts of how they experienced CBT in this RCT described physical weakness, apathy and poor concentration as reasons for struggling with therapy. Based on these findings, future research exploring whether fatigue is a barrier to therapeutic engagement is warranted. A challenge for this field is the conceptual issues with the construct of depression. For example, poor concentration is listed as a separate symptom of depression to fatigue (APA, 2013), but is experienced, based on our participants’ accounts, as intertwined. Furthermore, apathy may be part of the symptom experience of anhedonia in adolescents who are depressed, and adolescents with anhedonia describe fatigue (Watson, Harvey, McCabe, & Reynolds, 2020). Network analysis has begun to shed light on how symptoms are linked and inter‐related. For example, in a community sample of adolescents using the Children’s Depression Inventory, fatigue was most strongly associated with sleep disturbance (Mullarkey, Marchetti, & Beevers, 2019). To further clarify the conceptualization of fatigue as a depressive symptom in diagnosis, the dimensionality of fatigue should be considered in relation to its presence, severity and duration in medical and psychological disorders (Billones, Kumar, & Saligan, 2020).

Adolescents experienced uncertainty and apprehension when starting therapy. This finding is consistent with previous qualitative studies of therapy, including psychodynamic psychotherapy, which reported that adolescents communicated uncertain and cautious expectations of therapy (Midgley et al., 2016; Weitkamp, Klein, Hofmann, Wiegand‐Grefe, & Midgley, 2017). To tackle their initial uncertainty, adolescents have expressed a need to feel understood and accepted, which enabled them to open up more fully (e.g., Lavik, Veseth, Frøysa, Binder, & Moltu, 2018). Rogers (1957) emphasized genuineness of the therapist as a key component required to form a therapeutic relationship. This is consistent with our findings and with a previous study looking at depressed adolescents who had good outcomes from CBT (Wilmots, Midgley, Thackeray, Reynolds, & Loades, 2020). Wilmots et al. (2020) found that the therapist being perceived as genuine provided a sense of safety where adolescents felt able to open up. Therefore, adolescents may be particularly vulnerable during initial sessions before a therapeutic relationship is established (Binder, Moltu, Hummelsund, Sagen, & Holgersen, 2011).

It was also found that fatigued adolescents reported finding the exploration during the early sessions emotionally evocative. They struggled to cope with these difficult emotions and at times appeared to prefer to focus on self‐management. It is unclear why this was a common experience among our participants. Exploring adolescents’ current difficulties is an important stage of treatment which enables the therapy plan and goals to be established (Institute for Quality & Efficiency in Health Care, 2013). If fatigued adolescents struggle with the emotions which arise from discussing their difficulties in early sessions, they may be at risk of ending therapy prematurely (e.g., Wilson & Sperlinger, 2004). Further research is needed to identify whether struggling with emotional exploration during early sessions is a collective experience among fatigued adolescents receiving CBT for depression.

Adolescents experienced CBT as both helpful and unhelpful, with structure being helpful when they felt supported and viewed therapeutic goals as achievable. The structure of CBT appeared to provide reassurance about the therapeutic process. Adolescents expressed that structure enabled clear goal setting, an understanding of the changes therapy could provide and helped them navigate therapeutic tasks in manageable steps.

Adolescents who experienced CBT as providing safety through the therapeutic alliance and as being sufficiently flexible to meet their needs, (e.g., reduced session length) seemed to find therapy helpful overall. Bordin’s (1975) model extends the therapeutic alliance beyond the relationship between therapist and client to include agreement on the goals of therapy and agreement on therapeutic tasks (DiGiuseppe, Linscott, & Jilton, 1996). In our study, adolescents who felt that therapeutic tasks and goals were manageable or set collaboratively with the therapist found CBT helpful. Other participants, who felt therapeutic tasks were set unilaterally by the therapist or perceived therapeutic goals and tasks as unmanageable found therapy less helpful. This is consistent with Donnellan et al. (2013) who reported that the pace of a structured delivery is important in how therapy is experienced by adolescents receiving CBT for mental health issues. Our findings suggest that structuring activities can help for adolescents to gradually increase their physical activity, which may combat fatigue (Puetz, 2006).

CBT places importance on homework completion to help consolidate therapeutic gains between sessions and to ensure that improvement is maintained post‐treatment (Beck, 1979). However, adolescents frequently discussed difficulties engaging in therapeutic homework, including difficulties balancing CBT homework with schoolwork. This is consistent with previous research (e.g., Gaynor, Lawrence, & Nelson‐Gray, 2006). Similarly, Bru et al. (2013) reported that adolescents with depression described homework as helpful but effortful and time consuming. However, a few of our participants found reviewing CBT homework enabled them to monitor their progress, which appeared to encourage the adoption of helpful techniques (Kazantzis, Dattilio, Cummins, & Clayton, 2013).

Psychological therapies need to be adapted to adolescents’ developmental needs (Fuggle, Dunsmuir, & Curry, 2012). Adolescents’ cognitive abilities tend to increase with age, although there are many individual differences (Garber, Frankel, & Herrington, 2016). The formulation‐led approach of CBT allows therapists the flexibility to make developmentally appropriate adaptions whilst maintaining fidelity to the model. For example, the hot‐cross bun model (Greenberger & Padesky, 1995) can be used to help adolescents to identify connections between their thoughts, feelings and behaviours. Qualitative research has highlighted the importance of flexibility for adolescents accessing psychological services (Gibson, Cartwright, Kerrisk, Campbell, & Seymour, 2016). Our findings suggest ways in which flexibility may apply to depressed adolescents who are fatigued, including shorter sessions to alleviate difficulties concentrating, and ensuring rest days were not interrupted.

Most adolescents described CBT as facilitating change. For some, change was achieved through engaging in activity, which provided a sense of independence. Others implemented cognitive strategies and reflected on how therapeutic homework helped them to monitor and maintain positive change or described increased understanding of themselves or others.

Although CBT was experienced as challenging, most adolescents thought that it had helped by providing them with skills to identify and manage their thoughts and emotions. CBT typically not only involves cognitive restructuring but also encourages self‐management by teaching adolescents coping skills, including distraction techniques (Kennerly, 2016), which some adolescents reported using. Distraction can be useful in managing thoughts (Loades, Clark, & Reynolds, 2014) and studies have reported that some depressed adolescents found cognitive restructuring difficult to implement and preferred to alleviate symptoms by distraction (Bru et al., 2013). It may be that distraction is less effortful cognitively. However, distraction may become unhelpful if it is used as a safety behaviour as it does not allow adolescents to change unhelpful cognitions (Kennerley, 2016). Thus, offering adolescents different strategies to experiment with so that they can adopt those that work best for them is important for treatment.

### Limitations

Because we relied on analysing data that had been collected as part of a wider study, we were not able to purposively sample prospectively. This resulted in relying on one measure of fatigue within the K‐SADS rather than being able to use more extensive fatigue‐specific measures to select our sample, which could have strengthened representativeness. Additionally, the interview topic guide was designed to provide a broad overview of the adolescents’ therapeutic experience. It did not explicitly ask about fatigue, and we selected relevant segments to inform our analysis.

Our participants received therapy as part of a clinical trial, and this may have influenced their experience of CBT. For example, adolescents may have felt obligated to continue with therapy; similarly, research interviews about their experience of therapy would not normally take place and could have increased perceived support in treatment.

Based on our findings which suggest that fatigue is often an important experience for adolescents receiving CBT for depression, prospectively designed studies could focus more specifically on gaining further insight into how fatigued adolescents experience CBT. Future research could also explore the extent to which fatigue changed during CBT, which was beyond the scope of this study. It was also beyond the scope of this study to compare the experience of CBT in fatigued and non‐fatigued depressed adolescents. Therefore, some of the findings may also apply to non‐fatigued adolescents. Furthermore, reflexivity could have been improved through consultation with adolescents with lived experience of depression and fatigue. This could have enhanced the authenticity of the results and strengthened data interpretation.

### Implications for future research and clinical practice

Although NICE recommends addressing sleep problems in the context of depression, it makes no recommendations for treating fatigue in adolescent depression (NICE, 2019). Our study suggests that attending to fatigue as part of the treatment of depression in adolescents may be important, as fatigue appeared to impact on their therapeutic experience. Using fatigue screening questionnaires like the Chalder Fatigue Questionnaire (Chalder et al., 1993) or the Fatigue Associated with Depression Questionnaire (Matza, Phillips, Revicki, Murray, & Malley, 2011) could ensure that fatigue is appropriately assessed. Although, further work on the psychometric properties of these questionnaires in adolescents is required to ensure validity and reliability. Additionally, using fatigue‐specific tools alongside other measures could help distinguish fatigue from other depressive symptoms, such as apathy as part of anhedonia, which can also be assessed using the K‐SADS and more specifically using the Apathy Evaluation Scale (Marin, Biedrzycki, & Firinciogullari, 1991). However, more work is needed to understand the interconnections between symptoms like anhedonia and fatigue, which adolescents with depression have described as being linked (Watson et al., 2020). We also advise future researchers to explore contextual influences on fatigue and CBT for depression, including adolescents’ expectations of therapy and how adolescents understood their difficulties, (Midgley et al., 2015, 2016). Family, peer and cultural factors were also overlooked in this study due to the fatigue‐focused data selection from the transcripts.

As fatigue may impede therapeutic engagement and influence adolescents’ experiences of therapeutic activities and goals, it is important for clinicians to assess fatigue and adapt therapy as needed. For example, fatigued adolescents may need therapeutic support to gradually increase their activity and/or session length may need to be reduced. Additionally, fatigued adolescents may require clinicians to be flexible and supportive towards therapeutic homework assignments, which our participants found demanding. This exploratory study provides some preliminary indications of how treatment may need to be adapted for this population, but further research is required to investigate how fatigue may impact specific components of therapy.

### Conclusions

Fatigued adolescents appear to experience difficulties adjusting to CBT, taking part in sessions and engaging in homework, which may require sessions to be flexible to their needs. The structure of CBT can be helpful for adolescents to set and work towards goals and a structured approach to increasing activity may empower adolescents in between sessions. Making sure that the pace of sessions fits the individual’s needs, and establishing achievable shared goals appeared important.

This study has provided an initial insight into how depressed adolescents, who report fatigue, experience CBT. It provides preliminary indications of how treatment may need to be adapted to meet their needs. More research is needed to further understand the experience and the impact of fatigue on outcomes, and to refine psychological treatments to address fatigue, which can persist beyond therapy.

## Conflicts of interest

The authors declare that there is no conflict of interest.

## Author contribution

**Georgia Tanith Herring:** Conceptualization (equal); Data curation (equal); Formal analysis (equal); Methodology (equal); Project administration (equal); Writing – original draft (equal). **Maria Elizabeth Loades:** Conceptualization (equal); Funding acquisition (equal); Project administration (equal); Supervision (equal); Writing – review & editing (equal). **Nina Higson‐Sweeney:** Data curation (equal); Writing – review & editing (equal). **Emily Hards:** Supervision (equal); Writing – review & editing (equal). **Shirley Reynolds:** Writing – review & editing (equal). **Nick Midgley:** Data curation (equal); Writing – review & editing (equal).

## Data Availability

The data used in this study are owned and stored by the Anna Freud Centre.
